# Fluoroless endourological surgery for high burden renal and proximal ureteric stones: A safe technique for experienced surgeons

**DOI:** 10.1080/2090598X.2021.1901357

**Published:** 2021-03-18

**Authors:** Elias M. Ayoub, Ali Bourgi, Josee Alsouki, Sleiman Merhej, Pierre Conort

**Affiliations:** a Department of Urology, Hôpital Français Du Levant, Beirut, Lebanon; b Faculty of Medicine, Saint Joseph University, Beirut, Lebanon; c Department of Urology, Hôtel-Dieu De France, Beirut, Lebanon; d Department of Urology, Pitié Salpêtrière, Assistance Publique – Hôpitaux De Paris, Paris, France

**Keywords:** Ureteroscopy, urolithiasis, percutaneous, radiation protection

## Abstract

**Objective**: To describe the feasibility of treating proximal ureteric and renal stones using flexible ureteroscopy (fURS) or a double approach (mini-percutaneous nephrolithotomy [PCNL] + fURS) without any use of radiation.

**Patients and methods**: We retrospectively reviewed the data of all patients operated by one surgeon for retrograde endoscopic removal of renal and ureteric lithiasis performed between June 2015 and January 2019 in our institution. Patients with anatomical complexities, high-burden stone disease (diameter >20 mm), and medical comorbidities (anti-platelet drug administration) were included in our study. Outcomes analysed included complication rate, stone-free rate (SFR, defined as no residual stone >1 mm), and repeat procedure rate.

**Results**: In all, 183 consecutive URS for proximal ureteric and renal lithiasis were conducted. C-arm fluoroscope guidance was not required, not even in the complex cases. Simultaneous ultrasonography and fURS guidance was used in patients where the mini-PCNL approach was indicated. Lead aprons were not needed by the operating room staff in any of the operations. The SFR was 91.8% after the first procedure, with no Clavien–Dindo Grade III or IV complications.

**Conclusion**: Our present series shows clearly that the fURS and mini-PCNL approach under fURS control is a feasible and safe technique for experienced surgeons. Patients had a high SFR and no technique-related complications, with no additional risk of X-ray exposure. However, a prospective study is required to test the reproducibility of this technique.

**Abbreviations**: GMSV: Galdakao-modified supine Valdivia; ICRP: International Commission on Radiological Protection; KUB: plain abdominal radiograph of the kidneys, ureters and bladder; OR: operating room; PCNL: percutaneous nephrolithotomy; SFR: stone-free rate; UAS: ureteric access sheath; (f)URS: (flexible) ureteroscopy; US: ultrasonography

## Introduction

Conventional endourology has always been related to fluoroscopic guidance since its development in the mid-20th century. Goodwin et al. [1] were the first to describe percutaneous nephrostomy and antegrade pyelography in 1955 with X-ray guidance.

Fluoroscopy later gained more interest in the urology field with the introduction of ureteroscopy (URS) and percutaneous nephrolithotomy (PCNL) for the treatment of urinary lithiasis.

With improving endoscopic techniques, more complex cases are treated endoscopically with a higher risk of radiation exposure due to longer operating times [2]. Although the amount of radiation exposure during fluoroscopy for endourological interventions is not as high as diagnostic imaging, it still contributes to increased total body exposure and of course the effects of radiation are cumulative [3].

According to several studies, the prevalence of urinary lithiasis is 2–3% in general population [4], with a recurrence rate estimated at 30–40% within 5 years [5]. Based on these statistics, patients with urolithiasis are more likely to have several CTs during their lifetime with increased risk of other X-ray exposure during endourological procedures. These facts have caused a huge increase in medical radiation exposure, clearly described in a study conducted in the United States, which compared the annual per capita radiation exposure in 1980 and 2006 (0.54 vs 3.0 mSv respectively) [6,7].

Surgeons and operating room (OR) staff are also exposed to X-rays, with not only radiation exposure risk (eyes for example), but also direct and indirect morbidity related to the weight of the lead aprons worn during fluoroscopic-guided interventions. Therefore, the International Commission on Radiological Protection (ICRP) set a maximum occupational exposure limit of 50 mSv/year [8].

Several measures have been undertaken to restrict any unnecessary radiation exposure. The principle of ‘as low as reasonably achievable’ should be adopted when using fluoroscopy. In addition to the use of lead aprons, increased distance from the source, and parameters adjustment of the X-ray source protocols have been developed to decrease fluoroscopy time without altering patient outcome, surgery time or complexity [9].

Recent studies have described the feasibility of URS with minimal or no fluoroscopic guidance [10–13]. However, all the series reported, have limited stones size with limited numbers of patients.

In our present series, we describe the feasibility of 183 consecutive proximal ureteric and renal stones using flexible URS (fURS) or a double approach (mini-PCNL + fURS) without any use of radiation. Patients with high-burden stone disease (diameter >20 mm) and medical comorbidities (anti-platelet drug administration) were also included in the study.

## Patients and methods

We retrospectively collected the data of all patients operated for retrograde endoscopic removal of lithiasis between June 2015 and January 2019 by the same surgeon. Exclusion criteria included patients treated for upper tract malignancy (16 patients), ureteric strictures or malformations (21), distal ureteric lithiasis (45) and staghorn stones for whom PCNL was planned initially (34).

Patients with a large stone diameter were informed preoperatively of the optional indication of a mini-PCNL approach to improve stone fragmentation and extraction. Those patients were included in our study.

We recorded demographic and perioperative information including: age at time of surgery, gender, laterality, presence of preoperative ureteric stent, stone location, and mean operative time. Stone size was equivalent in our study to the largest cross-sectional diameter. In patients with of multiples stones, we calculated the total diameter by adding the largest cross-sectional dimension of each stone. Outcomes analysed included: complication rate, stone-free rate (SFR, defined as no residual stone >1 mm), and repeat procedure rate.

### Technical aspect

#### Preoperative

All patients had a preoperative non-contrast abdomino-pelvic CT. Reviewing the CT in bone window can give more information about the composition, the number, and the hardness (Hounsfield unit [HU] density) of the stones.

All patients were operated under general anaesthesia. Patients were placed in a lithotomy position. Patients with higher stone burden (largest cross-sectional diameter >20 mm, multiple renal stones) were placed in the Galdakao-modified supine Valdivia (GMSV) position for an eventual mini-PCNL approach if any difficulty was encountered to assure a stone-free status.

#### Perioperative

A rigid cystoscope is introduced to inspect the bladder and to remove an existing JJ stent (positioned earlier for patients presenting initially with obstructive pyelonephritis, acute renal failure or hyperalgesic renal colic). A 7-F ureteric catheter is used to introduce a 0.089-cm (0.035-inch) hydrophilic tip guidewire (Sensor™; Boston Scientific, Marlborough, MA, USA) up to the kidney. Using tactile feedback, the guidewire is gently advanced ~20 cm until feeling a little resistance, which indicates its intrarenal positioning. Insertion of the ureteric catheter on 20–25 cm can also assess the absence of stenosis. Based on our experience with the fluoroless technique, an approximate evaluation of the remaining extracorporeal length of the guidewire gives a further assessment of the correct guide’s position.

For proximal ureteric and intrarenal stones, an 8.5-F flexible digital uretero-renoscope (FLEX-XC™, Karl Storz Endoskope, Tuttlingen, Germany) is advanced over the guidewire to explore the ureter for any stricture or ureteric stones. For single and small renal stones, direct extraction with a Nitinol tipless stone basket (2.2 F N-Circle™; Cook Medical, Bloomington, IN, USA) is performed. For all other cases, a ureteric access sheath (UAS; Flexor™; Cook Medical) is gently positioned over the guidewire. A 12–14-F UAS is usually used. In case of resistance, the obturator is used to carefully dilate the ureter before retrying. A smaller diameter UAS (Flexor 10.7–12.7 F) is used if any resistance is still encountered.

A complete renal mapping is performed with localisation of all stones. Fragmentation or vaporisation is done with a holmium-laser fibre.

When a high stone burden is confirmed, after an attempt at lasering, it is decided whether the mini-PCNL approach should be implemented, as explained to the patient before the procedure. In these cases, preoperative CT was suspicious of this situation and the patient was installed in a GMSV position. Percutaneous access is performed under double simultaneous control: ultrasonography (US) and retrograde fURS. US can detect the fURS tip moving in the ideal calyx to be punctured. When the needle is introduced under US-guidance, the fURS is monitored during all the different steps, from needle insertion to the 16-F mini-PCNL sheath introduction in the calyx. A wire (Sensor; Boston Scientific) is introduced percutaneously, retrieved with the fURS down to the bladder using a tipless basket as a grasper. Successive dilatations are then performed over the guidewire under fURS control. The mini-nephroscope (Karl-Storz Endoskope) is then introduced to complete stone fragmentation and extraction. The aim is to remove all fragments of >1 mm. The fURS is used to review all the calyces and check the stone-free status. The mini-nephroscope ensures the position of a 7-F ureteric catheter introduced in the Flexor sheath and fixed to a Foley catheter for few hours. The mini-nephroscope is retrieved just behind the papilla and a haemostatic matrix (Floseal™, Baxter BioSurgery, Vienna, Austria) is used to close the percutaneous tract.

In case of a single access by fURS, a complete exploration of the renal cavities is performed to detect any residual fragments. Then the safety guidewire is repositioned in the superior calyx under direct vision, and the ureteroscope is gently retrieved alongside with the UAS in order to inspect the integrity of ureteric mucosa and to detect any residual ureteric fragments.

The UAS can be reintroduced in order to put in place a 7-F ureteric catheter. If double pigtail stenting is chosen, the stent is positioned under URS control (in women) or cystoscopic control (in men). A double pigtail stent is usually used when residual stones are present or when excessive ureteric oedema or ureteric perforation is suspected.

Use of lead aprons is not required by the surgeon, the assistant or the OR staff, although the C–arm fluoroscope is kept inside the operating room in case of any complication encountered during the operation, as required by the European Association of Urology (EAU) guidelines [14].

The stone-free state was confirmed in all patients by performing a renal US and a plain abdominal radiograph of the kidneys, ureters and bladder (KUB) 1 month after the endoscopic extraction.

## Results

Between June 2015 and January 2019, 183 consecutive URS for proximal ureteric and/or renal lithiasis were performed by the same surgeon in our institution. C-arm fluoroscope guidance was never required, not even in complex cases. US guidance was used for the mini-PCNL approach when indicated. Lead aprons were never used by the OR staff in any of the operations. Patients and stones characteristics are reported in Tables 1 and 2.

**Table 1.** Patients’ characteristics

Characteristic	Value
Age, years, mean (range)	47.3 (24–81)
Sex, % Female Male	45.9 53.1
Past medical history, % Recurrent lithiasis Cystinuria Cacchi–Ricci disease Chronic anti-platelet drugs	57.9 19 9 10

**Table 2.** Stones’ characteristics

Characteristic	Value
Side, % Right Left Bilateral	33.8 50.8 15.4
Size, mm, mean (range) >20 mm, %	16.7 (8–50) 26.2
Stone number, % Single Multiple	49.1 51.9
Previous stent, %	27.3
Localisation, % Upper calyx Middle calyx Lower calyx Pelvis Proximal ureter Multiple calyces	15.3 7.1 33.8 8.2 16.4 19.2

### Population studied

Patients included in this study were not candidates to any restriction related to their age, medical history or stone characteristics. The mean (range) age was 47.3 (24–81) years. Recurrent urinary lithiasis was encountered in 57.9% of patients, of whom 19 had cystinuria and nine medullary sponge kidney disease. In all, 10 patients were under chronic use of anti-platelet drugs, which were not discontinued before the operation; these patients were candidates for fURS only.

The mean stone size was 16.7 mm. A stone burden of >20 mm was noted in 26.2% of patients, and 54.6% of patients had multiple urinary lithiasis. Only 27.3% of patients had a previous stenting (most of whom presented initially with obstructive pyelonephritis or hyperalgesic renal colic).

### Procedure technical aspects

Almost all fURS were done through a UAS (Flexor 12/14 F in 74.9% of patients, Flexor 10.7/12.7 F in 15.8%, and no UAS in 9.3%).

In 62.8% of patients, postoperative drainage was a ureteric catheter left *in situ* for a maximum of 20 h postoperatively (removed at 06.00 hours on Day 1). A JJ stent was placed in 26.3% of patients. Only 10.9% had no postoperative renal drainage; these patients had a unique <7 mm calculus extracted without the use of UAS. The mean (range) surgical time was estimated at 88 (30–210) min. A longer operating time (120–210 min) was noted for patients (14.8%) who underwent the mini-PCNL approach related to an increased stone burden (20–50 mm).

### Postoperative

The SFR was 91.8% after the first procedure.

Complications were graded according to the Clavien–Dindo classification and there were no Grade III–IV (major complication requiring intervention) complications. Six patients had postoperative gross haematuria treated conservatively (those patients were informed of the high risk of bleeding due to chronic anti-platelet treatment). One patient with a small pelvic perforation, noted perioperatively, was managed by JJ stent placement for 2 months.

All patients were planned to be discharged on Day 1 in our routine practice, those who had gross haematuria were discharged on Day 2. Only 10 patients were operated on an ambulatory basis. In all, 12 patients needed a second procedure 1 month later due to residual fragments (Table 3).

**Table 3.** Peri- and postoperative outcomes

Characteristic	Value
UAS, % Flexor 12/14 F Flexor 10.5/12.7 F None	74.9 15.8 9.3
Operating time, min, mean (range)	88 (30–210)
Postoperative drainage, % Ureteric catheter JJ stent None	62.8 26.3 10.9
SFR, %	91.8
Complications, % Gross haematuria Pelvic perforation	3.3 0.5
Mini-PCNL approach, %	14.8
Ambulatory, %	5.5

## Discussion

Fluoroscopic guidance is widely used in endourological interventions for lithiasis (PCNL, shockwave lithotripsy, URS). Patients with recurrent urolithiasis are at higher risk of radiation exposure, especially during diagnostic imaging. A study showed that a patient presenting with renal colic is prone to an average of four radiographic imaging session in 1 year (1.7 CTs, 1.2 KUB, and an excretory urogram), with a total radiation exposure of 29.7 mSv [15].

During a standard URS, the patient’s median radiation exposure is estimated at 1.13 mSv, equivalent to a simple KUB [16]. On the other hand, a study conducted by Hellawell et al. [17] in 2005 showed that the surgeon receives a mean of 11.6 μGy during each fluoroscopic-guided endourological procedure. This could cause a yearly cumulative dose of 5.8 mGy in high-volume centres. When multiplied by the residency years and the surgeon’s career it could be equivalent to 17 CT scans. The ICRP recommends an annual occupational radiation exposure limit of ≤50 mSv/year [8]. With an increase of radiation exposure to the patient, surgeon and OR staff, there is a risk of increased morbidity such as cataracts, malignancy, and lead protection-related morbidity (e.g. back pain, neck pain) [18–20].

From a physiopathological perspective, there is no defined limit for radiation exposure morbidity. All ionising radiation has the potential to cause harm, especially cancer development. This risk could be categorised as a deterministic or stochastic effect. Whereas the first effect is related to an exposure above a certain threshold and increases with higher exposure (cardiological and dermatological effects) [17], the second is not related to a predefined limit and may be triggered with any ionising radiation exposure, such as cancer (leukaemia, lymphomas, solid cancers) [3,21]. A study conducted by Krupp et al. [22] in 2010, showed that about one in 1000 patients will develop cancer after fluoroscopic-guided URS.

Lead apron protection, which is usually heavy, has also been described to cause severe spinal injuries to surgeons and OR staff. Ross et al. [23] used the term ‘interventionalist’s disc disease’ to describe cardiologist skeletal modifications (multiple level disc herniation, cervical complaints). This was related to an increase in the number of missed days from work.

As fluoroscopy time is the simplest way to quantify operative radiation exposure, it was considered as the main factor in most studies and safety recommendations. Studies have shown that adopting minimal technical modifications can reduce perioperative radiation exposure by ~82% [24]: place the fluoroscopy unit image intensifier as close to the patient as possible, use of pulsed instead of a continuous fluoroscopy [25,26], performing a pre-fluoroscopy checklist [27], providing surgeons with feedback on their fluoroscopy usage after each procedure [28].

Other groups have developed awareness radiation programmes for residents, e.g. Safety, Minimisation and Awareness Radiation Training (SMART), which showed a decrease of 56% in fluoroscopy time [29]. Furthermore, a significant decrease of fluoroscopy time was noted with increasing resident experience, as shown by a study conducted in 2014 that followed the improvement of two residents during their first 2 years of residency (a 79% decrease in radiation time was noted) [30].

Reduced radiation fluoroscopy protocols (fluoroscopy mainly used for UAS insertion and complicated renal mapping and stent placement) have been implemented in some centres with reduced radiation exposure of up to 83% in some studies with similar SFRs and operating times compared to conventional URS [24,31].

In order to limit radiation exposure, other series described US-guided URS, based on its common use in pregnant women [32]. A randomised controlled trial was conducted in 2014 of 50 patients to compare conventional URS to US-guided URS. No differences were found in terms of duration, complications and SFR. Nonetheless, all patients included in that study had one ureteric stone of <8 mm [33]. Another study conducted by Singh et al. [34] reported a high success rate of >95% with US guidance for ureteric calculi of <10 mm. However, US guidance needs special expertise and specific equipment in the OR with limited studies concerning renal lithiasis.

Surgical and medical specialties have recently published several series omitting fluoroscopy from the conventional technique. Ablative cardiac interventions for extrasinusal tachycardia were performed with no fluoroscopic guidance in children, pregnant women, and obese patients [35].

In the urology field, some studies have noted the feasibility of URS with minimal or no fluoroscopic guidance. Nevertheless, these studies had small numbers of cases, with limited stone burden and clear restriction criteria for complex cases.

Mandhani et al. [10], performed in 2007, fluoroless URS for small distal ureteric stones. In all, 4% of the cases needed minimal fluoroscopic guidance. They also showed the feasibility of distal ureteric balloon dilatation under direct vision. Later, Tepeler et al. [11] described 93 cases of distal and proximal semi-rigid URS with a successful fluoroless technique in 92.4%, with no major complications. However, a KUB was obtained in all patients on day 1. Hsi et al. [12] published a series of 162 patients with renal and ureteric stones treated endoscopically: 75% did not require any fluoroscopy and 85% required a maximum of 2 s of fluoroscopy (for stent placement, median effective dose of 0.05 mSv).

In 2014, the feasibility of completely fluoroless URS was shown over 50 consecutive patients with no significant difference in complications rate, mean operative time, and repeat procedure rate. It was the first series to treat proximal and distal URS without any fluoroscopic guidance for renal mapping or stent placement. Nevertheless, patients had a lower stone burden than those operated by the conventional technique in the same institution [13].

In our centre, we started using the fluoroless technique in 2014 only for limited stone burden. Rapidly, all cases of ureteric or renal stones, even stones of >20 mm, were routinely performed by one surgeon without any use of fluoroscopic guidance. The mini-PCNL approach was needed for larger stones in order to increase the SFR in one session. Tactile feedback, fixed landmark and surgical experience are essential to perform this technique. Digital fURS and two video charts were the main improvement needed to offer the mini-PCNL approach option in the case of large stones.

In the present series, we report the outcomes of 183 consecutive cases, including complex cases with high burden stone disease (>20 mm) and even patients with chronic anti-platelet drugs administration. Fluoroscopy was not required in any of these cases. The mini-PCNL approach was performed under simultaneous US and fURS guidance (as described above). The SFR was 91.8%, very similar to other conventional URS studies [13].

Importantly, we noted that this technique was completely safe with minimal risk to the patient. In addition, the main benefit for the urologist and the OR staff with this fluoroless technique (besides the decrease in radiation exposure) was the opportunity of omitting lead aprons for protection, which is responsible for neck and back pain, reported as ‘interventionalist’s disc disease’ by Ross et al. [23].

Unfortunately, we could not perform a comparative study with conventional URS, as the fluoroless technique is performed by the referent stone surgeon of our centre, whereas the conventional technique is still used by fellows and residents for less complex cases.

Comparison of fluoroless surgery time with the conventional technique was not feasible, as the stone burden was variable in our present series with complex cases that needed further manipulations (balloon dilatation, percutaneous approach).

During our practice, we noticed that graduated guidewires would be a tool to help us establishing fixed and objective landmarks during fURS. Moreover, although three-dimensional US could facilitate our technique, especially when the mini-PCNL approach is needed, it remains a high cost technology with no studies published showing its benefit in endourology.

Although endourological interventions with no fluoroscopy decrease the amount of radiation in the OR, omit lead aprons usage, and ensure less morbidity to the surgeon and OR staff with less stressful time especially for pregnant women, it is important to mention that this technique was performed by one highly experienced surgeon in our department and could not be considered as the standard of care due to the eventual complications that could be caused by young or inexperienced surgeons e.g. ureteric and pelvic trauma or stent malpositioning.

## Conclusion

Our present series shows clearly that fluoroless URS, and the mini-PCNL approach under fURS control, was a feasible and safe technique in all cases in our study. Patients had a high SFR and no technique-related complications, with no additional risk of X-ray exposure. However, a prospective study is required to test the reproducibility of this technique.
